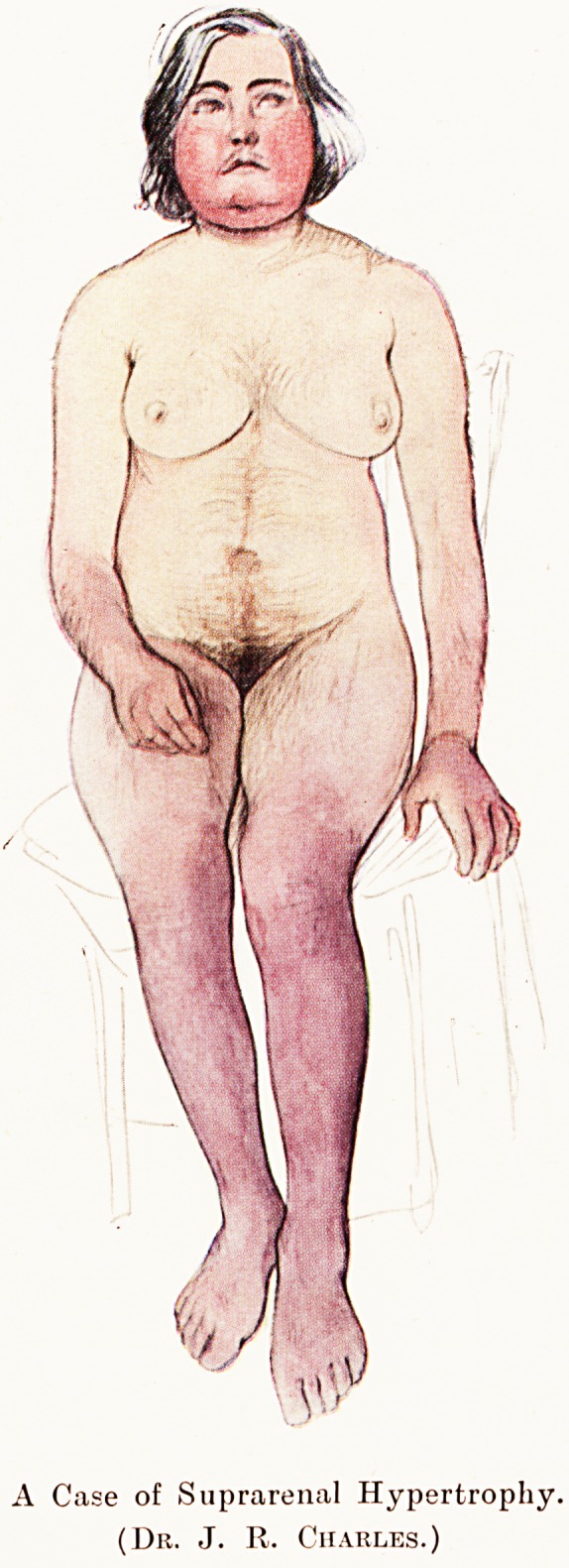# Suprarenal Hypertrophy

**Published:** 1932

**Authors:** J. R. Charles

**Affiliations:** Physician, Royal Infirmary, Bristol


PLATE I

A Case of Suprarenal Hypertrophy.
(Dr. J. R. Charles.)

PLATE II

A Case of Suprarenal Hypertrophy.
(Dr. J. R. Charles.)

### The Bristol Medico-Chirurgical Journal

*"Scire est nescire, nisi id me
Scire alius sciret."*

SUMMER, 1932.

### SUPRARENAL HYPERTROPHY.

BY

J. R. Charles, M.D., F.R.C.P.,

*Physician, Royal Infirmary, Bristol.*

Not many examples of suprarenal virilism treated successfully by operation are as yet on record. This particular one seems to deserve reporting, not only because of this, but also because of the unusual character of the lesion found. The following note is brief, but the illustrations which accompany it will, it is thought, make certain aspects of the disorder clearer than words could; particularly the cyanotic colour.

R. W., a female, aged 20, was sent to see me on 11th March, 1931, complaining of a growth of hair all over her body and face, with high blood-pressure. There was no history of any abnormality in the family. She had been perfectly well when young, and had no serious illness.

She first noticed that hair was growing excessively on her legs, arms and face in August, 1930, and that she was putting on weight very rapidly. About a month later she noticed a very considerable amount of hair growing on her body, especially the abdomen. Up to the time of admission it had been increasing in quantity, and was getting much more coarse. She developed so much hair on her face and upper lip that she was exceedingly worried by it, and used "Veet" to get rid of it. Her face got very much larger, and her colour became a deep purple, especially on the face, backs of arms, legs and back of neck. Her menstruation started when she was eleven years old, and she was quite regular until 1930, when from about January she had intervals of about two to four months, and before her visit she had had complete amenorrhœa for fourteen weeks. She had not complained of any aches or pains.

On examination, the hair that she described was very obvious. Her skin was very hard, dry and scaly, the papillæ being very prominent. The thighs showed a mottled red appearance. Below the knees both legs were a deep scarlet-purple, and the upper and posterior surfaces of both arms were of the same hue. There was also an area with a similar condition of purplish skin over the upper part of the back, extending from the sixth cervical to the fourth dorsal vertebræ. Nothing abnormal was found in the lungs, nervous system or urine, nor in the abdomen; no tumours were made out in the suprarenal areas. Her heart was rather rapid, and her systolic blood-pressure when first seen was 150, with a diastolic of 100. Her thyroid could not be felt. An X-ray of the skull showed no evidence of any pituitary lesion. Her blood sugar curve was normal, and Wassermann reaction negative. An X-ray of her kidneys after "Abrodil" showed nothing of importance, but there was a slight shadow on the left side. She was given thyroid gr. v. t.d.s. and a tablet of whole ovarian gland three times a day.

A diagnosis of suprarenal hypertrophy was made, and on 23rd April, 1931, an operation was performed by Mr. Walters, who found no evidence of any tumour. The kidney appeared to be normal, the right suprarenal was not palpable, but the left was felt, and there were adhesions between it and the spleen. He removed the left suprarenal gland, which showed hypertrophy, the weight of it being five grammes (normal = 3–4 grammes). She made a very satisfactory recovery, and was discharged on 25th May, still on thyroid treatment.

Her periods then returned for the first time since December, 1930, and remained regular for the next four months. She was re-admitted to the Infirmary on 10th September, 1931. At that time, in her own words, she said, "I was very much better. My colour was much less the deep purple, and was a brighter red. The texture of the hair was much finer." She was kept on thyroid gr. v. twice a day, and also given intra-muscular injections daily of 1 c.c. solution of anterior lobe of the pituitary, and also 1 c.c. ovarian extract.

As the hair on her face was causing her very considerable mental distress, she had an exposure to X-rays. The hair began to fall out in ten days, and in three weeks her face was quite clear, and much softer in texture. The hair on her arms and abdomen was treated with hydrogen peroxide, and became very much less visible. She was discharged again on 15th December, very greatly improved. Her periods ceased for a short time when she was in the Infirmary, but returned on going home, and she has been quite regular since. She continued to attend as an out-patient, and treatment with thyroid and ovarian extract was continued. Early in April this year she had a second application of X-rays to her face.

A very large number of observations have been made on her blood-pressure, which was not found on subsequent investigations to be as high as when she was first seen, typical readings in November and December, 1931, being 126 up to 134, but her weight has gradually increased, and on 5th April, 1932, she weighed 10 st. 2 lb. Her face is still large, and her cheeks still somewhat purplish-red in colour, but the disfiguring coloration has considerably diminished. Her complexion now is that of a red peach. The coarse hair has entirely disappeared from her face, a light-coloured, soft, downy hair taking its place. The hair on the abdomen has not diminished; her back, however, is still red, her legs also are red, but much less so than when she was first seen, and her arms are practically normal. She is very much brighter and happier in disposition than when first seen.

## Figures and Tables

**Figure f1:**
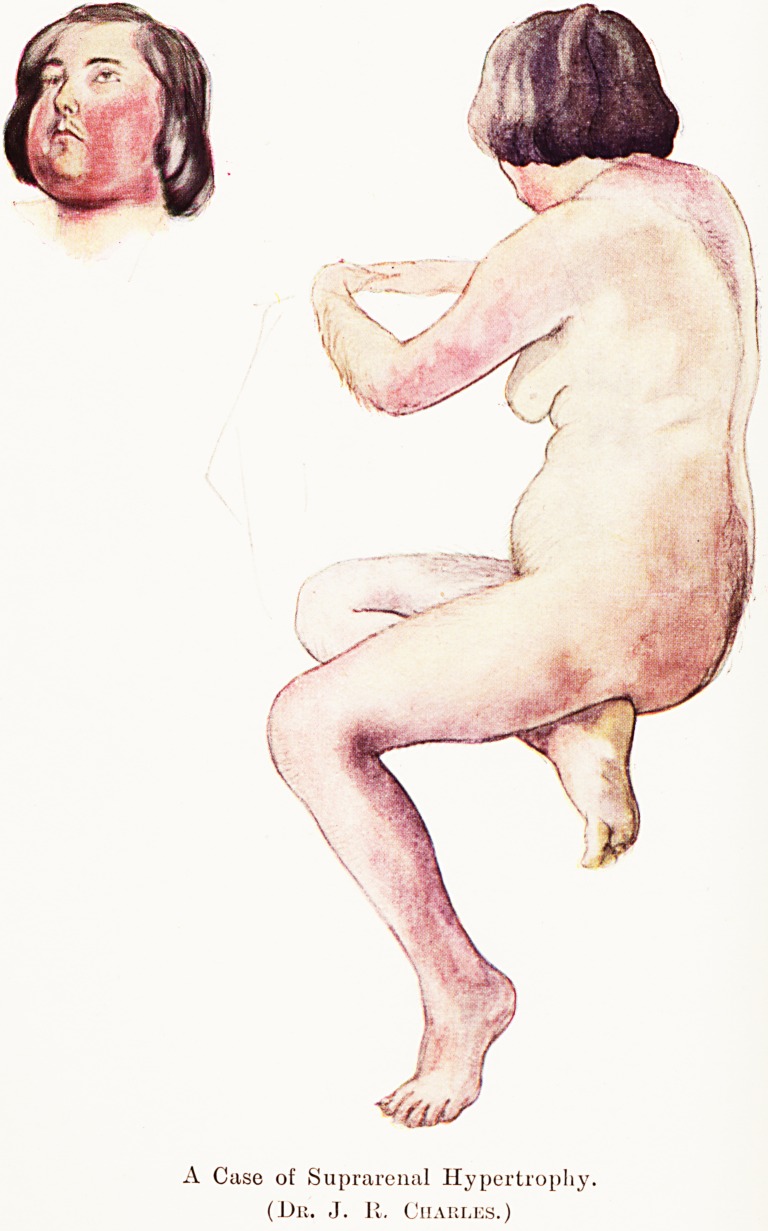


**Figure f2:**